# Numerical Investigation on Anti-Explosion Performance of Non-Metallic Annular Protective Structures

**DOI:** 10.3390/ma16247549

**Published:** 2023-12-07

**Authors:** Xiaobing Bian, Lei Yang, Tao Wang, Guangyan Huang

**Affiliations:** 1 State Key Laboratory of Explosion Science and Technology, Beijing Institute of Technology, Beijing 100081, China; 3220205032@bit.edu.cn (X.B.); yangl@bit.edu.cn (L.Y.); wang_tao@bit.edu.cn (T.W.); 2 Beijing Institute of Technology Chongqing Innovation Centre, Chongqing 401120, China

**Keywords:** blast protection, non-metallic annular structures, numerical simulation, UHMWPE

## Abstract

Explosive shock wave protection is an important issue that urgently needs to be solved in the current military and public security safety fields. Non-metallic protective structures have the characteristics of being lightweight and having low secondary damage, making them an important research object in the field of equivalent protection. In this paper, the numerical simulation was performed to investigate the dynamic mechanical response of non-metallic annular protective structures under the internal blast, which were made by the continuous winding of PE fibers. The impact of various charges, the number of fiber layers, and polyurethane foam on the damage to protective structures was analyzed. The numerical results showed that 120 PE fiber layers could protect 50 g TNT equivalent explosives. However, solely increasing the thickness of fiber layers cannot effectively enhance the protection efficiency. By adding polyurethane foam in the inner layer, the stress acting on the fiber could be effectively reduced. A 30 mm thick polyurethane layer can reduce the equivalent stress of the fiber layer by 41.6%. This paper can provide some reference for the numerical simulations of non-metallic explosion protection structures.

## 1. Introduction

In today’s world, the spread of explosive terrorist incidents poses a serious challenge to human civilization, regional peace and national security [1]. The disposal of various types of military explosives, homemade explosives, unexploded ordnance and other explosives is complex and arduous. The safety protection of explosives is the most important part of the explosive ordnance disposal process, which usually involves the use of explosion-proof containers of a certain strength to restrain the hazards generated by explosives [2,3].

Unlike traditional metal or concrete materials [4,5,6,7], the structurally weak materials represented by fibers have shown great promise for internal blast mitigation. The structurally weak materials can convert the blast energy into their own kinetic and internal energy and are almost completely converted into small soft particles after loading, which have virtually no secondary damage compared to rigid materials such as metals [8].

In recent years, the structurally weak materials such as water, fibers and foams have been widely used in the field of blast mitigation [9,10,11]. Zhou et al. [9] showed that the annular foam and liquid protective structure, which is a protective structure with promising applications, can significantly reduce the peak pressure of the explosion at a certain distance. Batra et al. [10] investigated the three-dimensional transient deformation of unidirectional fiber laminates subjected to blast loading. Ply splitting was found to be the dominant damage mode, absorbing 80% of the shock wave energy. Fallah et al. [11] compared the deformation of mild steel and ultra-high molecular weight polyethylene (UHMWPE, referred to as PE) fiber with the same face density under blast loading. It was found that the mild steel had ruptured after loading, and PE fibers could reduce the local deformation by 30%.

PE fiber is one of the best ballistic performance fibers available, which has been widely used in bulletproof vests, bulletproof helmets, bulletproof panels, and other equipment designs [12,13]. The common PE fiber structures mainly include fiber filaments, fiber cloths, and fiber plates. The single-layer PE fiber cloth is generally made of four layers of PE fibers orthogonally hot-pressed, and the ratio of fiber to resin is about 4:1 [14].

**Figure 1:**
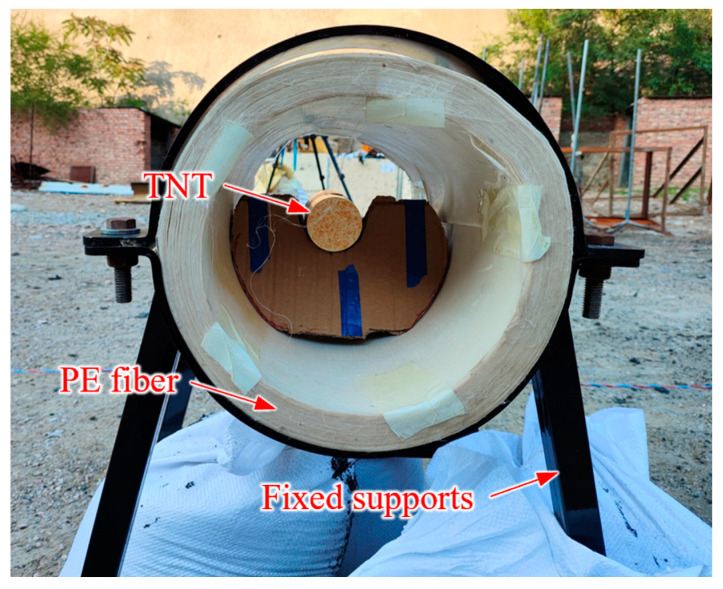
Photograph of the previous experimental setup.

**Figure 2:**
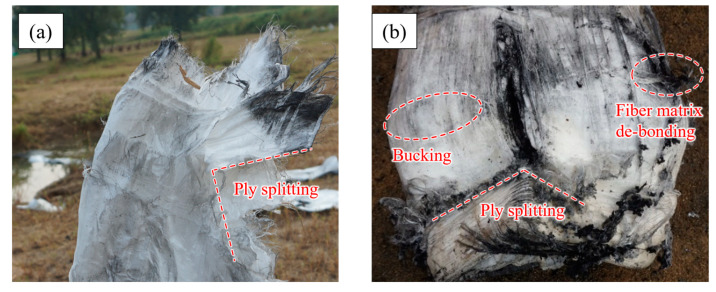
Photograph of the PE fibers after loading: (**a**) overall structure, (**b**) outermost layer.

However, according to our previous blast tests (Figure 1), visible damage or even disintegration occurred in the PE fiber after loading. The structure of the PE fiber after loading is shown in Figure 2, which included various failure modes such as ply splitting, bucking, and fiber matrix de-bonding, etc. Hence, adding a cushioning material in front of the fiber layer was a viable way to reduce structural damage [15,16,17,18,19]. Karagiozova et al. [15] analyzed the deformation mechanism of FML (Fiber-metal laminate) based on the composition of glass fiber panels and aluminum panels under blast loading. Sitnikova et al. [16] conducted a series of experiments and numerical simulations to analyze the dynamic response of FML under blast loading. The fiber layer was found to have fractured locally and a petal-shaped perforation was formed in the middle of the target plate. Although the FML sandwich composite structure was effective in reducing fiber deformation, the secondary damage could be caused by the broken pieces when the aluminum plate was shattered. Thus, it was necessary to design a weak material such as foam to act as a buffer layer for the fibers and evaluate their blast mitigation performance, which has hardly been studied before.

This paper carried out a series of numerical simulations of the non-metallic composites under blast loading. The blast mitigation effect of PE fibers was comparatively investigated before and after the addition of polyurethane (PU) foam. The results of this investigation showed that the added PU foam on the inside of the PE fibers can effectively reduce the stress of the fiber, avoiding the direct disintegration of the fiber structure under the internal blast loading. The main innovation of this paper is the use of foam “weak material” as a cushioning layer for fibers, and greatly improving the protection of fiber materials against blast shock waves, which has been little investigated before. It is expected to be able to provide a certain reference for the engineering design of explosion-proof structures.

## 2. Numerical Simulation Description

The numerical simulation conditions in this paper are shown in Table 1. The inner diameter and height of the annular PE fiber were 200 mm, and 400 mm, respectively. Working conditions 1–3 were mainly used to compare the protective effect of the same number of fiber layers under different trinitrotoluene (TNT) equivalents. The working conditions 1, 4 and 5 were to compare the effects of different fiber layers on protection under the same charge (200 g TNT). The working conditions 1, 6 and 7 were to compare the effects of the increased PU foam energy-absorbing layer on the protective structure under the same charge (200 g TNT) and the same number of fiber layers (120 layers of PE fibers).

### 2.1. Numerical Model and Simplification

To further investigate the buffering effect of PU foam on PE fiber, a series of numerical simulations were conducted using explicit dynamics solvers in LS-DYNA. The Structured Arbitrary Lagrange-Euler (S-ALE) method was used to define fluid-structure coupling contact, which referenced the immersed boundary method (IBM) [20,21]. Compared with the traditional ALE method, the S-ALE method can avoid leakage and improve computing efficiency [22]. The pinball segment-based contact penalty formulation has been used to define the contact force between the foam and the PE fibers without tangential friction.

**Figure 3:**
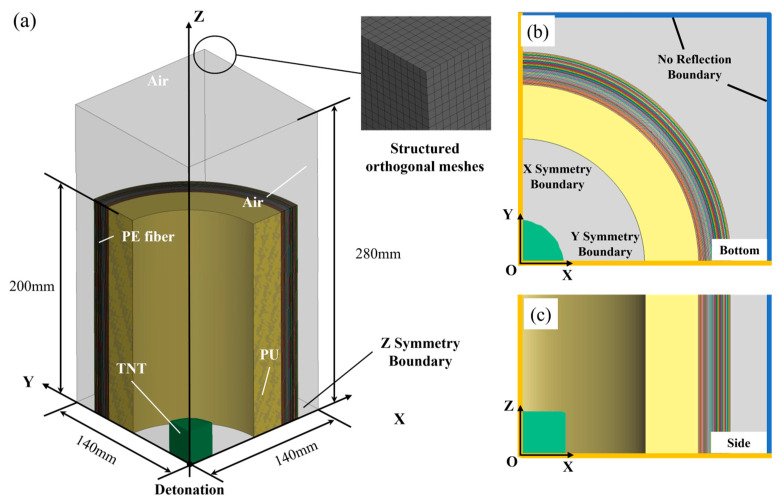
Numerical simulation model of the PE fiber and PU. (**a**) 1/8 of 3D model. (**b**) Bottom view of the numerical model (X-Y plane) with the boundary conditions. (**c**) Side view of the numerical model (X-Z plane).

As shown in Figure 3, the overall model was constructed using a 1/8 symmetric model, with the detonation point set at the center. Corresponding to this, three symmetry boundary conditions were applied to the *X* = 0, *Y* = 0, *Z* = 0 planes and no reflection boundary conditions were applied to other planes (Figure 3b,c).

The S-ALE calculation area was divided using 2 mm structured orthogonal meshes with a side length of 140 mm and a height of 280 mm. The sizes of TNT, PU foam and PE fibers varied under different working conditions, and Figure 3a showed only one of them. The PE fiber bulk density and surface density were 970 g/m^3^ and 150 g ± 5 g/cm^2^, respectively. The thickness of a single fiber layer was 0.15 mm. According to the relevant literature, combining four layers of 0.15 mm thickness PE fibers into one layer of 0.6 mm thickness fibers had little effect on the deformation of the structure at a high strain rate. In order to further simplify the calculations, four layers of fibers were equated to one layer [23].

The PU foam, PE fiber and Euler regions were meshed in numerical simulations. The total number of PU foam (30 mm thickness) elements was 100,500, and the total number of nodes was 109,888. The total number of PE fiber (30 layers) elements was 256,200 and the total number of nodes was 524,172. The element size of the Euler region was 2 mm, and the total number of elements was 70 × 70 × 140 = 686,000. The mesh was automatically generated using Hypermesh meshing software version 2019, with all elements being hexahedral and having a size of 2 mm. The thickness of the PE section was 0.6 mm, and the thickness direction was meshed as a single layer. The official LS-DYNA theory manual states that the Lagrange elements and Euler elements with the same size were recommended to avoid S-ALE algorithm crashes [24]. Thus, the TNT and air domains were adopted as S-ALE meshed with a size of 2 mm in this model.

### 2.2. Material Model

The standard Jones Wilkins Lee (JWL) equation of state was used to describe the mechanical behavior of TNT detonation products, with parameters derived from Lawrence Livermore National Laboratory [25]:

(1) $$P=A\left(1-\frac{\omega }{R_{1}V}\right)e^{-R_{1}V}+B\left(1-\frac{\omega }{R_{2}V}\right)e^{-R_{2}V}+\frac{\omega E}{V\prime }$$

where *A*, *B*, *w*, *R*_1_, *R*_2_ are the equation of state constants of the explosives.

The Linear Polynomial equation of state was used to describe the impact response behavior of the air [26]:

(2) $$P=C_{0}+C_{1}\mu +C_{2}\mu^{2}+C_{3}\mu^{3}+(C_{4}+C_{5}\mu +C_{6}\mu^{2})E_{0}$$

where *μ* = *ρ*/*ρ*_0_ − 1 is the ratio of the current density to the initial density. $C_{0}=C_{1}=C_{2}=C_{3}=C_{6}=0$, $C_{4}=C_{5}=\gamma -1$. For an ideal gas, γ=1.4. *E*_0_ is the internal energy per unit volume. The specific parameters are shown in Table 3.

**Figure 4:**
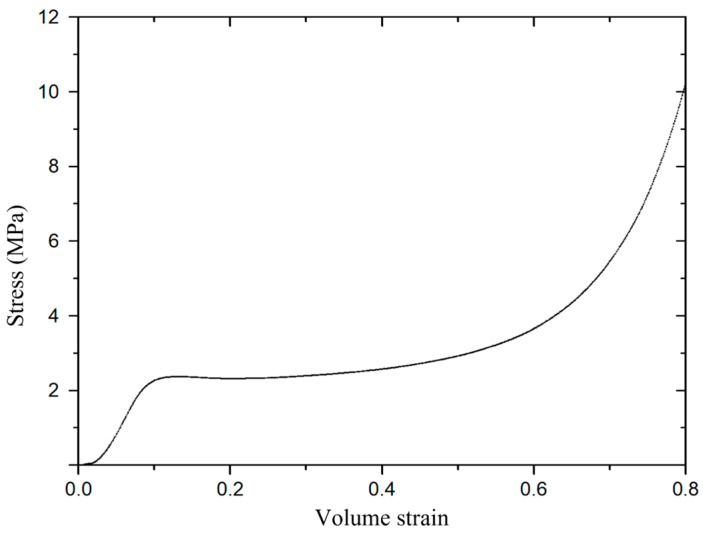
Volume strain and stress curve of the PU foam.

The PU foam material model was adopted as Crushable Foam with the parameters as shown in Table 3 [9]. It should be noted in particular that a failure criterion based on maximum principal strain (ε_MXEPS_) was used in the simulations. The volumetric strain and stress relationship is shown in Figure 4.

The PE fiber material was various anisotropic material. Considering that the fibers were wound molding, there was no connection between faces. Hence, there was no need to set up face-to-face bound contact. The composite failure material model was used in the LS-DYNA. The linear relationship of stress-strain was expressed as [27]:

(3) $$\left(\begin{array}{l} \varepsilon_{a}\\ \varepsilon_{b}\\ \varepsilon_{c}\\ \gamma_{bc}\\ \gamma_{ca}\\ \gamma_{ab} \end{array}\right)=\left(\begin{array}{cccccc} \frac{1}{E_{a}} & -\frac{v_{ba}}{E_{b}} & -\frac{v_{ca}}{E_{c}} & 0 & 0 & 0\\ -\frac{v_{ab}}{E_{a}} & \frac{1}{E_{b}} & -\frac{v_{cb}}{E_{c}} & 0 & 0 & 0\\ -\frac{v_{ac}}{E_{a}} & -\frac{v_{bc}}{E_{b}} & \frac{1}{E_{c}} & 0 & 0 & 0\\ 0 & 0 & 0 & \frac{1}{G_{cb}} & 0 & 0\\ 0 & 0 & 0 & 0 & \frac{1}{G_{ca}} & 0\\ 0 & 0 & 0 & 0 & 0 & \frac{1}{G_{ab}} \end{array}\right)\left(\begin{array}{c} \sigma_{a}\\ \sigma_{b}\\ \sigma_{c}\\ \tau_{bc}\\ \tau_{ca}\\ \tau_{ab} \end{array}\right)$$

where *E*, *G* and *v* are the modulus of elasticity, shear modulus, and Poisson’s ratio of the material, respectively. *ε*, *γ*, *σ* and *τ* are the strain, shear strain, positive stress and shear stress of the fiber, respectively. The following table *a*, *b* and *c* represent longitudinal, transverse and normal directions, respectively.

Five fiber material failure models and nine strengths were presented in Table 2, including three shear strengths (*S*_ab_, *S*_ac_, *S*_bc_), three compressive strengths (*C*_a_, *C*_b_, *C*_c_) and three tensile strengths (*T*_a_, *T*_b_, *T*_c_) [28]. The corresponding parameters are shown in Table 3.

## 3. Numerical Calculations and Analysis of Results

### 3.1. Effect of Different Explosive Equivalents

**Figure 5:**
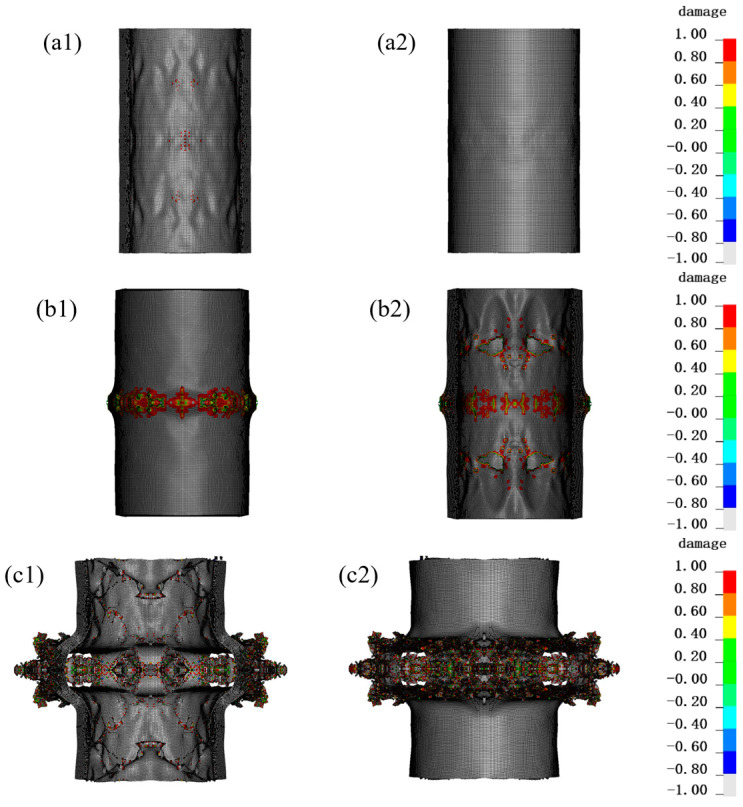
The damage of protective structure under different equivalent explosives. The legend represents the extent of damage to the material, where red (1) represents complete damage to the material and white (−1) represents no damage. (**a1**) Inner side, (**a2**) outer side of the PE fiber within 50 g TNT; (**b1**) Inner side, (**b2**) outer side of the PE fiber within 100 g TNT; (**c1**) Inner side, (**c2**) outer side of the PE fiber within 200 g TNT.

As shown in Figure 5, under the 50 g TNT internal blast loading, the fiber protection structure had no significant deformation. There was only a little damage to the innermost layer, which mainly concentrated in the center of the explosion. It could be basically considered that the structure was able to effectively protect against 50 g TNT explosives. Under the 100 g TNT internal blast loading, the inner and outer layers of fiber protection incurred some damage, which mainly concentrated in the center of the explosion. Hence, the structure under the 100 g TNT charge had a certain risk. Under the conditions of the 200 g TNT, the protective structure incurred a large fragmentation. The outermost layer was torn, which has been completely ineffective.

**Figure 6:**
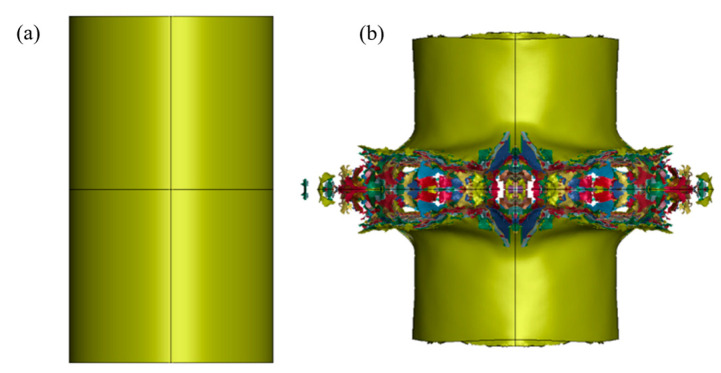
Comparison of protective structure heights before and after the explosion. Height of protective structure (**a**) before explosion, (**b**) after explosion.

**Figure 7:**
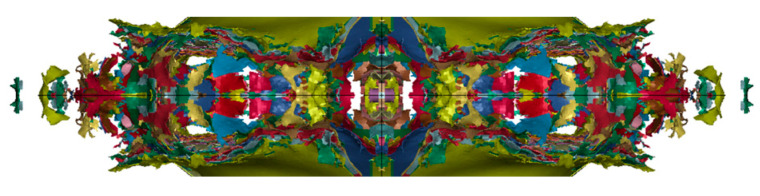
Crushing condition of the middle structure.

Numerical simulation showed that under the action of the explosion shock wave, the height of the protective structure could be reduced to the middle, as shown in Figure 6. For the 200 g TNT charge, its height before the explosion was 400 mm. After the explosion, the height was reduced to about 360 mm, a 10% height reduction. This was due to the large outward deformation of the middle protective layer under the action of the shock wave, as shown in Figure 7.

**Figure 8:**
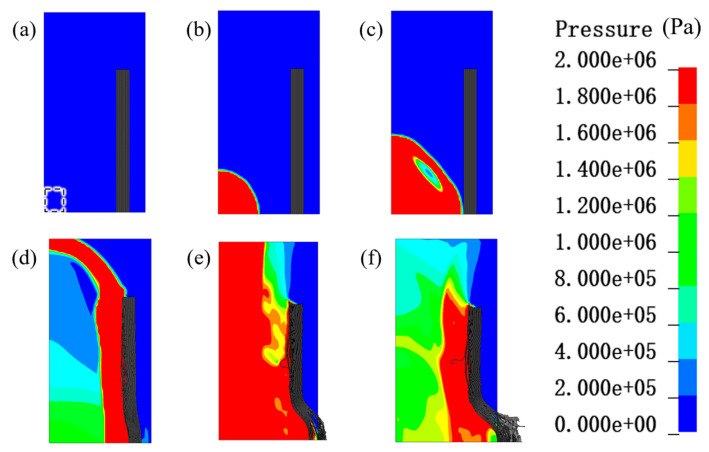
Time history of the interaction between shock wave and PE fiber: (**a**) Initial state at 0 ms, (**b**) blast wave propagation at 0.01 ms, (**c**) shock waves on fibers at 0.02 ms, (**d**) partial fiber failure at 0.07 ms, (**e**) massive fiber failure at 0.2 ms, (**f**) outermost layer destruction at 0.5 ms.

The time history of the blast shock wave and fiber interaction was shown in Figure 8. At 0.02 ms, the blast shock wave reached the fiber layer. At 0.07 ms, some overall deformation occurred, and some fibers were broken. At 0.2 ms, a large number of fibers failed and broke off. At 0.5 ms, the middle part has been completely broken and the protective structure has lost its protective ability.

### 3.2. Effect of Different Fiber Layers

**Figure 9:**
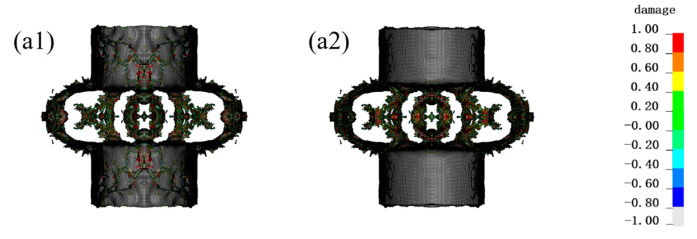

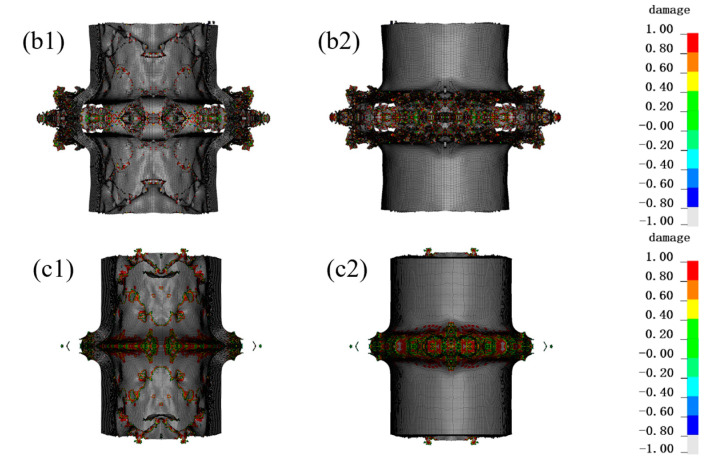
The effect of different fiber layers on protection. The legend represents the extent of damage to the material, where red (1) represents complete damage to the material and white (−1) represents no damage. (**a1**) Inner side, (**a2**) outer side of the 80-layer PE fiber; (**b1**) Inner side, (**b2**) outer side of the 120-layer PE fiber; (**c1**) Inner side, (**c2**) outer side of the 160-layer PE fiber.

**Figure 10:**
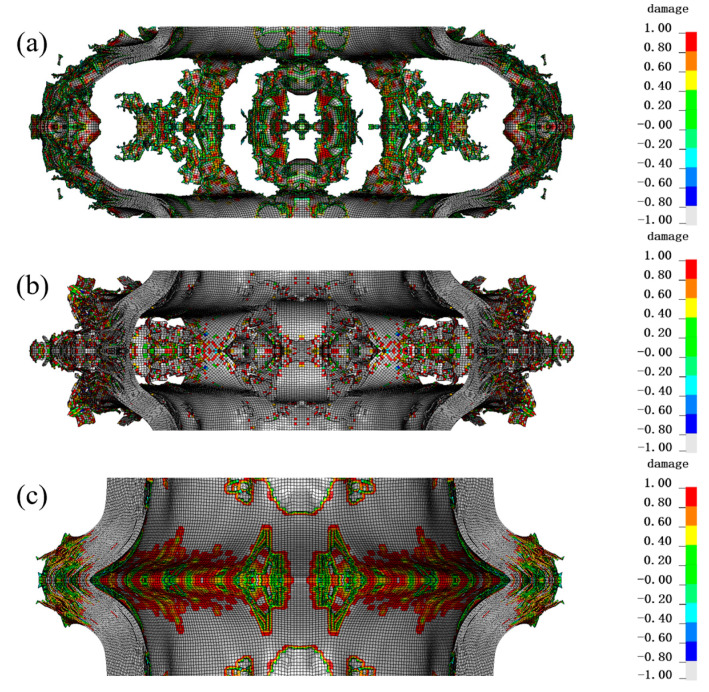
Damage situation of central parts with different fiber layers under blast loadings: (**a**) 80 layers of PE fibers, (**b**) 120 layers of PE fibers, (**c**) 160 layers of PE fibers. The legend represents the extent of damage to the material, where red (1) represents complete damage to the material and white (−1) represents no damage.

The dynamic mechanical response of 80-layer, 120-layer and 160-layer PE fibers under 200 g TNT charge is compared in Figure 9. And Figure 10 shows the damage of different fiber layers under blast loadings. Under the action of 200 g TNT charge, 80 layers of PE fibers were obviously deformed and broken. Of PE fibers, 120 layers were broken to a relatively small extent, and 160 layers were broken to the smallest extent, but the inner layer and the outermost layer were also broken. It can be seen that increasing the number of fiber layers can improve the protective effect to a certain extent, but cannot completely realize the explosion protection.

The shock wave generated by the explosion first acted in the innermost layer of PE, and the stress waves continued to propagate. In the continuous winding of the dense structure, the stress wave attenuation was small. A shock wave in the outermost layer of PE formed a reflected stress wave, resulting in the outermost fiber structure two times the stress state. Therefore, the damage generally occurred in the innermost and outermost layers of the fiber structure, and in some cases, the damage in the middle layer was smaller.

### 3.3. Effect of the PU Foam

Considering that increasing the number of PE layers did not effectively improve the stress state of the overall protective structure, the pressure peak of the inner PE fiber layer must be reduced to effectively reduce the damage. The PU foam was an excellent shock wave absorbing material, which could be placed inside the fiber structure to provide a cushioning effect.

**Figure 11:**
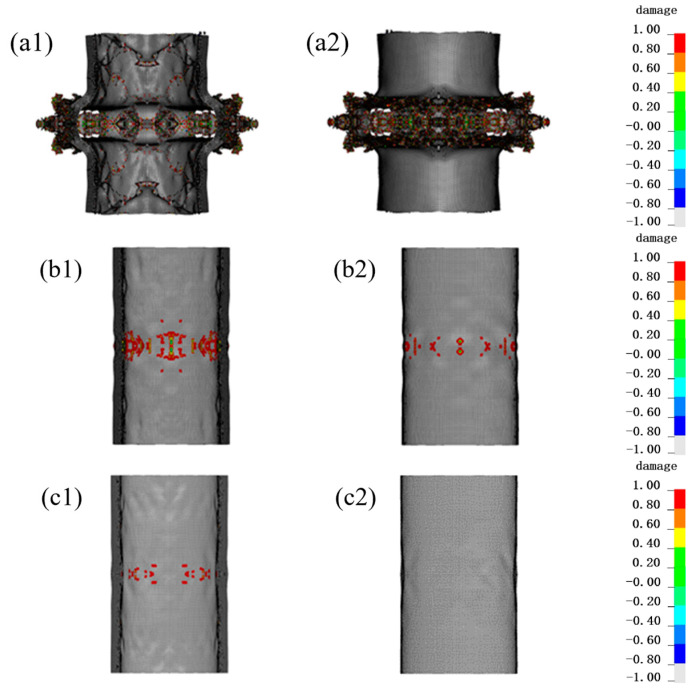
Damage situation of PE fiber structure after adding PU foam protective layer. The legend represents the extent of damage to the material, where red (1) represents complete damage to the material and white (−1) represents no damage. (**a1**) Inner side, (**a2**) outer side of the PE fiber without PU foam; (**b1**) Inner side, (**b2**) outer side of the PE fiber with 20 mm PU foam; (**c1**) Inner side, (**c2**) outer side of the PE fiber with 30 mm PU foam.

As shown in Figure 11, there was a significant reduction in the deformation of PE fiber after adding 20 mm PU foam, with only a little damage in the inner and outer layers. After adding 30 mm PU foam, there was almost no damage in the outermost layer of fibers.

**Figure 12:**
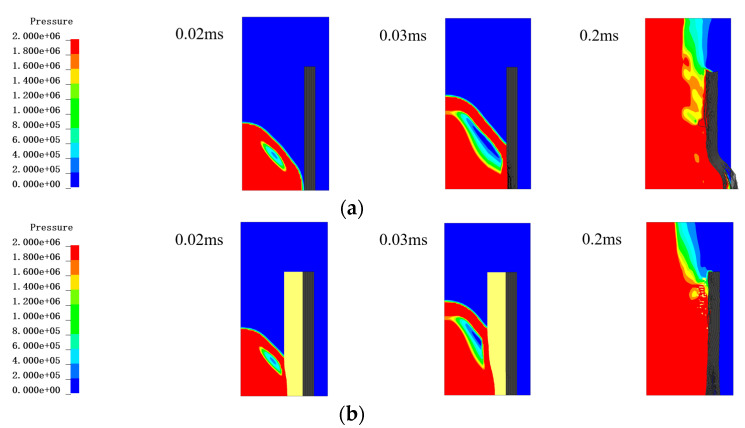
Comparison of the pressure contours in the Euler region (including air and TNT) in different conditions. The figure only showed the pressure of air and TNT without showing the fiber and foam. The unit of the pressure is Pa. (**a**) The PE fiber without PU foam at 0.02 ms, 0.03 ms and 0.2 ms; (**b**) The PE fiber with 30 mm PU foam at 0.02 ms, 0.03 ms and 0.2 ms.

The protective conditions of single fibers and 30 mm PU foam/fibers are compared in Figure 12. The yellow area in Figure 12b exclusively represents the PU foam material, excluding any indication of pressure. In the no PU foam condition, at 0.02 ms, the shock wave acted directly on the PE fibers, creating a large stress and a large number of fibers failing. At 0.03 ms, the stress wave continued to propagate to the outer layer, forming a reflection in the outermost layer. In contrast, under the 30 mm PU foam protection condition, at 0.02 ms, the shock wave acted on the PU foam layer and transmitted inward to a certain extent, and there was no crushing of fibers in the outermost layer. At 0.03 ms, a large amount of PU foam crushing occurred, absorbing a large amount of energy, while the foam in contact with the fiber generated a certain amount of stress wave propagation to the outer layer.

**Figure 13:**
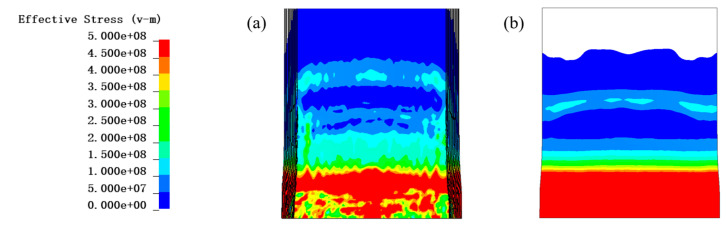
Contour diagram of effective stress of PE fiber without PU foam at 0.5 ms: (**a**) inner layer, (**b**) outer layer. The unit of the effective stress is Pa.

**Figure 14:**
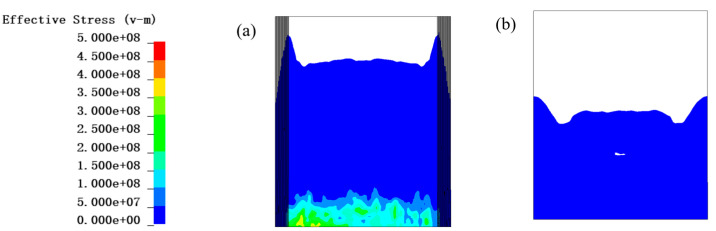
Contour diagram of effective stress of PE fiber with 30 mm PU foam at 0.5 ms: (**a**) inner layer, (**b**) outer layer. The unit of the effective stress is Pa.

Comparing Figure 13 and Figure 14, it can be found that at 0.05 ms, under the condition of no PU foam protection, the area of higher effective stresses in the fiber layer (≥500 MPa) was much larger than that of the 30 mm PU foam protection layer. Under the condition of no protection, the effective stress in the outermost layer of the fiber was greater than that in the innermost layer.

**Figure 15:**
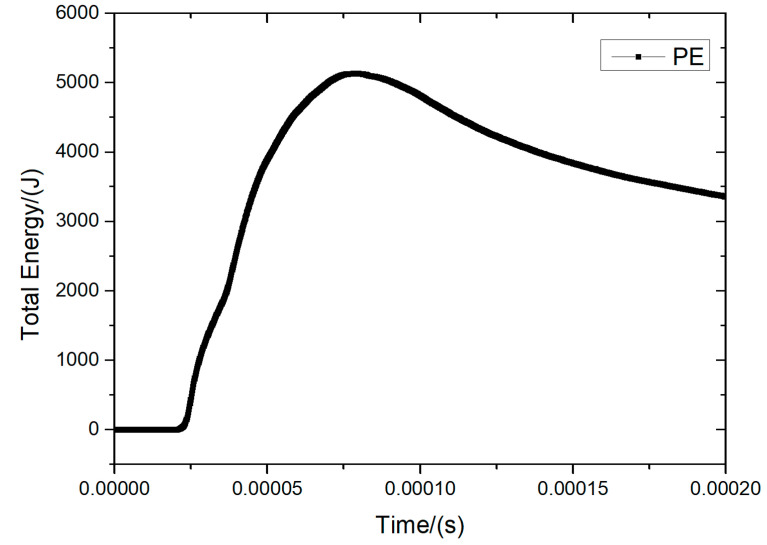
Total energy history curve of PE fiber layers without PU protection.

**Figure 16:**
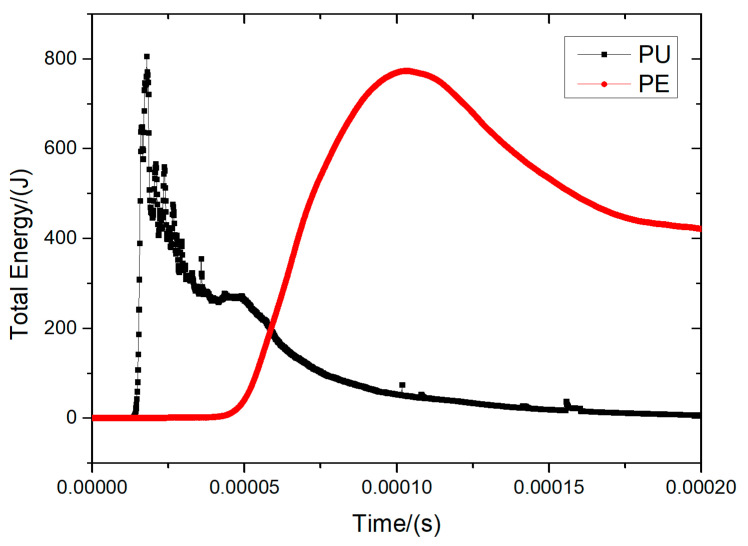
Energy history curve of 30 mm PU foam protective structure.

As shown in Figure 15 and Figure 16, comparing the PU foam (PU) and PE-only protection conditions at a thickness of 30 mm, the PU foam absorbed a significant amount of energy during the period of 0 to 0.05 ms. Thus, the total energy acting on PE fibers was significantly reduced.

**Figure 17:**
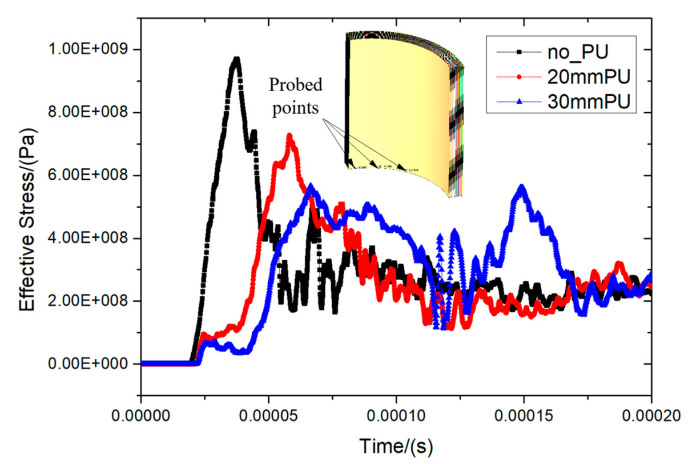
Effective stress history curves of fibers with different foam buffer layers.

Comparing no PU (no_PU), 20 mm thickness PU (20 mm PU), and 30 mm thickness PU (30 mm PU), the effective stress in the PE fiber layer is shown in Figure 17. It is important to note that Figure 17 shows the effective stress in the PE fiber, while Equation (2) calculates the shock wave overpressure in the air. Thus, it is reasonable that there is a large difference between them. The probed points were chosen to be three cells at the bottom, and the equivalent stress data from the three cells were averaged for comparison.

For the no PU foam, 20 mm PU foam, and 30 mm PU foam condition, the peak equivalent stress was 970 MPa, 727 MPa, and 566 MPa, respectively. As shown in Table 4, the 30 mm thickness of PU was able to significantly reduce the peak equivalent stress in the fiber layer by 41.6%.

## 4. Conclusions

In this paper, the numerical simulation of the non-metallic annular structures was investigated. By comparing the effects of different explosive equivalents, fiber layer thicknesses, and the PU foam on the protective structure, the numerical simulation analysis showed that:

(1) With the increase of explosive equivalents, the fiber protective structure under the same conditions was more likely to be broken. For this condition (inner diameter of 200 mm, length of 400 mm ring fiber structure, PE fiber layer number of 120 layers), the ring fiber could protect 50 g TNT bare explosives. For 100 g and above TNT charge, the annular fiber inner and outer layers would be torn, and there was a protection gap;
(2) The fiber thickness for bare explosives protection had less impact, and simply increasing the thickness of the fiber did not significantly improve the tearing situation;
(3) The inner layer increased 30 mm thickness PU foam material, and the PU foam can effectively absorb the shock wave energy. It reduced the inner layer of fiber on the equivalent stress of 41.6%, thus significantly reducing the tearing of PE fibers by shock waves.

The work in this paper for the non-metallic protective structures under the internal blast provided a numerical method reference. The next step of the work will focus on the protection structure for the blast test research.

## Figures and Tables

**Table 1:** Typical numerical simulation conditions.

Working Conditions	Protective Structure	Mass of TNT Charge
1	120 layers of PE fiber	200 g (50 mm diameter, 63 mm height)
2	120 layers of PE fiber	100 g (40 mm diameter, 50 mm height)
3	120 layers of PE fiber	50 g (30 mm diameter, 44 mm height)
4	80 layers of PE fiber	200 g (50 mm diameter, 63 mm height)
5	160 layers of PE fiber	200 g (50 mm diameter, 63 mm height)
6	120 layers of PE fiber (with 20 mm PU)	200 g (50 mm diameter, 63 mm height)
7	120 layers of PE fiber (with 30 mm PU)	200 g (50 mm diameter, 63 mm height)

**Table 2:** Failure model and criterion of the PE fiber.

Failure Model	Criterion
In-plane tensile failure	${\left(\frac{\sigma_{a}}{T_{a}}\right)}^{2}+{\left(\frac{\tau_{ab}}{S_{ab}}\right)}^{2}+{\left(\frac{\tau_{ac}}{S_{ac}}\right)}^{2}\geq 1$ ${\left(\frac{\sigma_{b}}{T_{b}}\right)}^{2}+{\left(\frac{\tau_{ab}}{S_{ab}}\right)}^{2}+{\left(\frac{\tau_{ac}}{S_{ac}}\right)}^{2}\geq 1$
Through-thickness tensile failure	${\left(\frac{\sigma_{c}}{T_{c}}\right)}^{2}+{\left(\frac{\tau_{ac}}{S_{ac}}\right)}^{2}+{\left(\frac{\tau_{bc}}{S_{bc}}\right)}^{2}\geq 1$
Through-thickness shear failure	${\left(\frac{\sigma_{a}}{T_{b}}\right)}^{2}+{\left(\frac{\tau_{ac}}{S_{ac}}\right)}^{2}\geq 1$, ${\left(\frac{\sigma_{b}}{T_{b}}\right)}^{2}+{\left(\frac{\tau_{bc}}{S_{bc}}\right)}^{2}\geq 1$
Longitudinal compression failure	${\left(\frac{\sigma_{a}}{C_{a}}\right)}^{2}\geq 1$
Through-thickness and transverse compressive failure	${\left(\frac{\sigma_{b}}{S_{ab}+S_{bc}}\right)}^{2}+\left[{\left(\frac{C_{b}}{S_{ab}+S_{bc}}\right)}^{2}-1\right]\frac{\sigma_{b}}{\left|C_{b}\right|}+{\left(\frac{\tau_{ab}}{S_{ab}}\right)}^{2}+{\left(\frac{\tau_{bc}}{S_{bc}}\right)}^{2}\geq 1$ ${\left(\frac{\sigma_{c}}{S_{ac}+S_{bc}}\right)}^{2}+\left[{\left(\frac{C_{c}}{S_{ac}+S_{bc}}\right)}^{2}-1\right]\frac{\sigma_{c}}{\left|C_{c}\right|}+{\left(\frac{\tau_{ac}}{S_{ac}}\right)}^{2}+{\left(\frac{\tau_{bc}}{S_{bc}}\right)}^{2}\geq 1$

**Table 3:** Material model parameters used for TNT, Air, Pu foam and PE fiber.

TNT material parameters [25]						
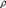 (kg/m^3^)	*A* (Pa)	*B* (Pa)		*R*_1_	*R*_2_	*w*
1.63	3.738 × 10^11^	3.747 × 10^9^		4.15	0.9	0.35
*D* (m/s)	*P*_CJ_ (Pa)					
6930	2.1 × 10^10^					
Air material parameters [26]						
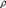 (kg/m^3^)	*C*_4_		*C*_5_		*E*_0_ (Pa)	
1.225	0.4		0.4		2.5 × 10^5^	
Foam material parameters [9]						
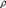 (kg/m^3^)	*E* (Pa)	*v*_r_		*T*_sc_ (Pa)	Damp	ε_MXEPS_
200	3.195 × 10^8^	0.001		5.3 × 10^6^	0.1	0.05
PE material parameters [27,28]						
*E*_a_ (Pa)	*E*_b_ (Pa)	*E*_c_ (Pa)		*v*_ba_	*v*_ca_	*v*_cb_
34.257 × 10^9^	34.257 × 10^9^	5.1 × 10^9^		0	0.013	0.013
*G*_ab_ (Pa)	*G*_ca_ (Pa)	*G*_cb_ (Pa)		*T*_a_ (Pa)	*T*_b_ (Pa)	*C*_c_ (Pa)
0.1738 × 10^9^	0.5478 × 10^9^	0.5478 × 10^9^		1.25 × 10^9^	1.25 × 10^9^	1.9 × 10^9^

**Table 4:** Comparison of equivalent stress peaks with different protective layers.

Protective Structure	Peak Equivalent Stress (MPa)	Comparison
No PU foam	970	0
20 mm PU foam	727	−25.1%
30 mm PU foam	566	−41.6%

## Data Availability

Considering that there is still more work to be done in the future, we have not chosen to publish all the data in this article.
